# Principles to promote social equality across the cancer trajectory: A group concept mapping study

**DOI:** 10.2340/1651-226X.2025.44738

**Published:** 2025-11-19

**Authors:** Maria Aagesen, Eva E. Wæhrens, Pernille Bidstrup, Gunn Ammitzbøll, Hanne Tønnesen, Eva Kjeldsted, Susanne O. Dalton, Karen la Cour

**Affiliations:** a Occupational Science, User Perspectives and Community-based Interventions, Department of Public Health, University of Southern Denmark, Odense, Denmark; b The Parker Institute, Copenhagen University Hospitals Bispebjerg – Frederiksberg, Frederiksberg, Denmark; c Psychological Aspects of Cancer, Danish Cancer Institute, Copenhagen, Denmark; d Department of Psychology, University of Copenhagen, Copenhagen, Denmark; e Danish Research Center for Equality in Cancer (COMPAS), Naestved, Denmark; f Cancer Survivorship, Danish Cancer Institute, Copenhagen, Denmark; g Department of Clinical Oncology and Palliative Care, Zealand University Hospital, Naestved, Denmark; h WHO-CC, Clinical Health Promotion Centre, the Parker Institute, Bispebjerg-Frederiksberg Hospital, Copenhagen University, Copenhagen, Denmark

**Keywords:** Oncology, social vulnerability, inequality in health, concept mapping

## Abstract

**Background and purpose:**

Social inequality is a growing problem throughout the cancer trajectory. Since 2019, the Danish Research Center for Equality in Cancer (COMPAS) has therefore, through seven work packages developed and tested various methodologies, approaches, and interventions to promote social equality in cancer from diagnosis to end of life. This study aimed to synthesize the knowledge generated across the work packages to provide guiding principles for promoting social equity across the cancer trajectory.

**Material and methods:**

A group concept mapping study was conducted in Denmark between February and June 2023. Twenty-two employees from all COMPAS work packages brainstormed ideas on how to promote social equality across the cancer trajectory. Fourteen participants subsequently sorted and rated the ideas by importance. Multidimensional scaling analysis and hierarchical cluster analysis were used to generate a cluster rating map outlining principles for promoting social equality in cancer. These principles were validated by 10 participants during an in-person validation meeting. Discussions from both the brainstorming and validation meeting were recorded, transcribed verbatim, and analysed.

**Results:**

Eight principles comprising 162 ideas were identified. Four principles focused on the patient-provider level: (1) Person-centred approach, (2) Supportive interventions targeting vulnerable patients, (3) Communication, and (4) Screening for vulnerability. Four addressed the organizational and policy level: (5) Skills development and implementation, (6) Coherence across, (7) Organizational and cultural factors, and (8) Transportation and accessibility.

**Interpretation:**

Integrating these principles into future research and clinical practice may support efforts to reduce social inequities across the cancer trajectory.

## Introduction

Social inequality persists throughout the cancer trajectory, from diagnosis and treatment to rehabilitation, palliative care, and end of life, representing a growing challenge in cancer care globally [1–4]. Low socio-economic position (SEP), typically defined as low income, short or no education, and living alone, is associated with poorer outcomes across the cancer continuum [5, 6]. For example, the incidence of several cancers is higher among individuals with low SEP. Further, they are more often diagnosed at a later stage, offered less treatment, and experience higher mortality rates compared to those with higher SEP [7, 8]. Even when adjusting for stage at diagnosis and comorbidity, individuals with low SEP still have lower odds of receiving standard treatment in some cancer types, for instance, both curative and palliative treatment for lung cancer, as well as bone marrow transplantation for acute lymphoblastic leukaemia [9, 10].

Similar patterns of inequality extend to cancer rehabilitation and palliative care, where individuals with higher SEP have greater access to and are more likely to engage in these services compared to those with lower SEP [11–15]. Danish population-based studies have found that longer education is associated with an increased likelihood of referral to rehabilitation services [12], that married individuals are more likely to engage with a specialist palliative care team compared to unmarried individuals [16]. Further, a structured review also found inequalities in access to palliative care among vulnerable groups diagnosed with cancer, indicating that these individuals receive lower quality palliative care [17]. These disparities may explain why a Danish population-based study of 2 to 12-year cancer survivors found that those with shorter education were more likely to report compromised health-related quality of life compared to those with longer education [18].

Since equal access to healthcare is a fundamental principle of the Danish welfare system, the evident social inequality in cancer trajectories has prompted an increased focus on promoting equality in cancer care over the past decade in Denmark [19]. In response, the Danish Research Centre for Equality in Cancer (COMPAS) was established in 2019 to explore and identify ways to minimize social inequality in cancer [20]. COMPAS employs a multidisciplinary approach and consists of a core centre and, from the outset, seven work packages, each addressing different aspects of inequality by employing various methodologies to promote social equity across the cancer care continuum [20].

This study aimed to synthesize the knowledge generated across the core centre and the work packages to provide guiding principles for promoting social equity across the cancer trajectory.

## Material and methods

### Participants

Participants were eligible if they were or had been researchers, Ph.D. or master’s students, clinicians, or collaborators in one of COMPAS’ work packages as described in Table 1. Purposive sampling was used. Participant information on sex, profession, workplace, work package affiliation and role, and publications related to COMPAS were gathered.

**Table 1:** Overview of the aims of COMPAS work packages.

Work page number	Name	Aim
Research Centre Core	Research Centre Core	To coordinate and promote research activity, collaboration and strategy across work package.
1	What is at stake?	To evaluate whether implementing a structured facilitation plan applied before, during, and after curative treatment for acute myeloid leukaemia by a dedicated facilitator team can reduce the incidence of serious infections, treatment delays, and premature treatment. discontinuation among patients with leukaemia.
2	Change the system not the patient	To investigate how social inequality in cancer outcomes arises by exploring how patients with limited social support, low education, or comorbidity as well as their relatives and healthcare professionals perceive and engage with care processes from diagnosis through treatment, rehabilitation, and palliative care.
3	Navigation through complex treatment	To develop individual support targeted socially vulnerable patients.
4	Against All Odds	To evaluate the efficacy of intensive SNAP (Smoking, Nutrition, Alcohol, and Physical activity) intervention among patients with cancer and risky lifestyle in randomized controlled trials, cohorts, and interviews.
5	Community-based rehabilitation and palliative care for socially vulnerable people with advanced cancer	To describe the rehabilitative and palliative trajectories of socially vulnerable patients with advanced cancer, and to develop and feasibility test a generic model to support existing cancer rehabilitation and palliative care services for this population.
6	Prepare instead of repair	To develop and test the effects of a prehabilitation program with exercise during neoadjuvant chemotherapy.
7	Treatment across	To optimize implementation of National Guidelines for Palliation.

### Study design and procedures

To address the study aim we combined Group Concept Mapping (GCM) with deepening group reflections on the generated ideas and resulting clusters [21, 22]. GCM is a structured, mixed-methods approach for gathering and analysing stakeholder perspectives involving a preparation phase followed by five phases: (1) brainstorm, (2) sorting and labelling, (3) rating, (4) generation of a cluster rating map, and (5) validation [21, 22].

Phase One was conducted both in person and online, while Phases Two to Four were online, and Phase Five was in person. The Concept System® GroupWisdom™ software: Concept Systems, Inc. Copyright 2004–2020; all rights reserved (hereafter: GroupWisdom™) software supported the online phases [23]. The study was conducted from February to June 2023, after nearly 5 years of COMPAS activity.

### Preparation phase

A focus prompt was piloted online among three researchers from COMPAS to ensure clarity and alignment with the study’s aim. No revisions were needed after the pilot test.

### Phase one; brainstorm

At the in-person meeting, participants were introduced to the study and GCM methodology. Subsequently, they individually submitted ideas in response to the focus prompt, ‘How can social equality be promoted in the cancer trajectory?’, drawing on studies from the COMPAS project. Ideas were collected using Padlet. Participants then discussed their ideas in groups, and reflections were audio recorded. They could also add new ideas after the group discussion.

Those unable to attend in person participated via GroupWisdom™ software, reviewing existing ideas, and contributing additional ones. After brainstorming, ideas were reviewed in preparation for Phases Two and Three by the first, second, and last author. Ideas with multiple meanings were split, and redundancies removed by consensus. The final set of ideas was re-imported into GroupWisdom™.

### Phases two and three; sorting, labelling, and rating of importance

Participants received an e-mail with instructions for sorting, labelling, and rating tasks, along with a GroupWisdom™ link. They sorted ideas from Phase One into piles, labelling each pile with the name they thought was most appropriate. They were not permitted to sort all ideas into one pile and each pile had to contain more than one idea. Later, they rated the importance of each idea on a four-point ordinal scale: 1 being ‘Not important’, 2 being ‘Somewhat important’, 3 being ‘Important’, and 4 being ‘Very important’. To ensure data quality, data from each participant in Phases Two and Three were included only if over 75% of ideas were sorted, piles were labelled, and no more than five ratings were missing.

### Phase four; generating a cluster rating map

Based on data from Phases One to Three the GroupWisdom™ software was used to generate a cluster rating map through statistical analyses, detailed further in the data analysis section.

### Phase five; validating

All participants were invited to an in-person meeting to review the cluster rating map. They received materials, including a list of ideas organized by clusters, a cluster map, a point rating map, and a cluster rating map [21]. Individually, they assessed idea placement and cluster labels, suggesting changes only when clearly needed. Reflections were then discussed in plenary and small groups to reach consensus. All discussions were audio recorded.

### Data analysis

Based on approved sorting and rating date from Phases Two and Three, Phase Four involved multidimensional scaling analysis using GroupWisdom™, producing a stress value (acceptable range: 0.20–0.36) to assess goodness of fit [24]. A hierarchical cluster analysis then generated multiple cluster solutions, with the most informative one selected. Cluster labels were suggested based on participant input in Phase Two, and a cluster rating map was created to show the importance of ideas by cluster height.

After the validation meeting, each cluster was summarized, and a median from the importance rating was calculated for each cluster. To deepen the understanding of the cluster content, the first author reviewed transcripts and audio recordings of the group reflections multiple times. These initial interpretations and elaborations were then discussed and refined in collaboration with the last author and the full author team.

### Ethical considerations

According to Danish law, ethical and data protection approval were not required, as the study involved no medical interventions or sensitive data [25]. Participants received study information and were informed about the right to withdrawal. All participants provided verbal informed consent.

## Results

Twenty-two people participated in the study, representing all seven COMPAS work packages and the core centre. They included 17 women and five men from diverse professional and academic backgrounds – ranging from master’s students to professors and spanning fields such as medicine, psychology, occupational- and physio therapy, nursing, and public health. As shown in Table 2, the group had collectively published 22 research papers at the time of the study, which informed the data collection and analysis.

**Table 2:** Published papers forming the bases of the participants’ knowledge of social inequality in cancer (*n* = 22).

Title	Design	Work package	Cancer trajectory
‘There’s so much wrong with me. I’ve just gotten a little sick’: Syndemic cancer experiences among people struggling with homelessness and severe substance use.	Qualitative, partly longitudinal, interviews	1	Treatment and survivorship
Who are the vulnerable lung cancer patients at risk for not receiving first-line curative or palliative treatment?	Population-based study	3	Treatment
Barriers and facilitators to national guideline implementation for palliative cancer care in a Danish cross-sectoral healthcare setting: A qualitative study of healthcare professionals’ experiences.	Qualitative focus and individual interviews	7	Palliative care
Neo-train: study protocol and feasibility results for a two-arm randomized controlled trial investigating the effect of supervised exercise during neoadjuvant chemotherapy on tumour response in patients with breast cancer.	Study protocol for a randomized controlled trial	6	Treatment
Patient-Related Characteristics Associated with Treatment Modifications and Suboptimal Relative Dose Intensity of Neoadjuvant Chemotherapy in Patients with Breast Cancer – A Retrospective Study.	Retrospective Study	6	Treatment
10-year nationwide trends in incidence, treatment patterns, and mortality of patients with myelodysplastic syndromes in Denmark.	Population-based cohort study	2	Treatment and follow-up
Nurse navigation, symptom monitoring and exercise in vulnerable patients with lung cancer: feasibility of the NAVIGATE intervention.	Feasibility study	3	Treatment
The gold standard program (GSP) for smoking cessation: a cohort study of its effectiveness among smokers with and without cancer.	Cohort study	4	Treatment and survivorship
The use and timing of rehabilitation and palliative care to cancer patients, and the influence of social vulnerability – a population-based study.	Population-based study	5	Rehabilitation and palliative care
Social vulnerability among cancer patients and changes in vulnerability during their trajectories – A longitudinal population-based study.	Longitudinal population-based study	5	Treatment and survivorship
NAVIGATE: improving survival in vulnerable patients with lung cancer through nurse navigation, symptom monitoring and exercise – study protocol for a multicentre randomised controlled trial.	Protocol for a multicentre randomised controlled trial	3	Treatment
Patients’ experiences of the COVID-19 pandemic and the change to telephone consultations in cancer care. Support Care Cancer.	Qualitative individual interviews	Core	Treatment and follow-up
Positive predictive values of haematological procedure codes in the Danish National Patient Registry-A population-based validation study.	Population-based validation study	2	Treatment and follow-up
Socioeconomic position and clinical outcomes in patients with myelodysplastic syndromes: A population-based cohort study.	Population-based cohort study	2	Treatment
STRONG for Surgery & Strong for Life – against all odds: intensive prehabilitation including smoking, nutrition, alcohol, and physical activity for risk reduction in cancer surgery – a protocol for an RCT with nested interview study (STRONG-Cancer).	Protocol for a randomized controlled trials	4	Treatment
Cancer rehabilitation and palliative care for socially vulnerable patients in Denmark: an exploration of practices and conceptualisations.	Grey literature study	5	Rehabilitation and palliative care
Identification of socially vulnerable cancer patients – development of a register-based index (rSVI).	Register-based study	5	Survivorship
A population-based survey of patients’ experiences with teleconsultations in cancer care in Denmark during the COVID-19 pandemic.	Population-based survey	Core	Treatment and follow-up
A qualitative study of mechanisms influencing social inequality in cancer communication.	Observations and qualitative interviews	1	Treatment
The Danish Myelodysplastic Syndromes Database: Patient Characteristics and Validity of Data Records.	Validation study	2	Treatment
Rehabilitation and palliative care for socioeconomically disadvantaged patients with advanced cancer: a scoping review.	Scoping review	5	Rehabilitation and palliative care
Longer distance to specialized treatment centres does not adversely affect treatment intensity or outcomes in adult acute myeloid leukaemia patients. A Danish national population-based cohort study.	Population-based cohort study	2	Treatment and follow-up

Work packages: 1 = What is at stake?, 2 = Change the system not the patient, 3 = Navigation through complex treatment, 4 = Against All Odds, 5 = Community-based rehabilitation and palliative care for socially vulnerable people with advanced cancer, 6 = Prepare instead of repair, and 7 = Treatment across, Core = Research Centre Core staff.

### Group concept mapping data and qualitative data

**Figure 1:**
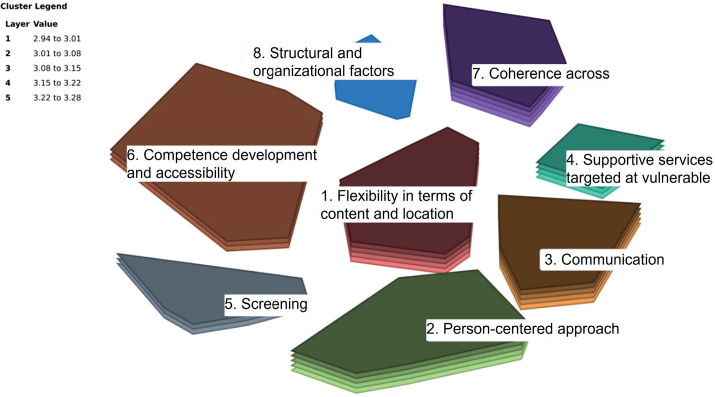
Cluster rating map.

In Phase One, 22 participants generated 152 ideas. After removing redundancies and separating ideas that contained multiple ideas, 162 unique ideas remained for subsequent phases. In Phase Two, 14 participants sorted and labelled the ideas; 13 rated them in Phase Three. All data met inclusion criteria and were included in the analysis in Phase Four. In this phase, multidimensional scaling involved 17 iterations and revealed a stress value of 0.30, indicating results within the acceptable range and thus interpretable. Cluster solutions from five to 10 clusters were considered by the first, second, and last author. An eight-cluster solution was selected for generating the cluster rating map (Figure 1), further examined at the validation meeting.

At the validation meeting, the 10 participants reached consensus on relocating 84 ideas (52%). This process involved deleting one cluster, constructing a new one, and renaming four existing clusters to better reflect their content, for example, renaming ‘Screening’ to ‘Screening for vulnerability’.

**Figure 2:**
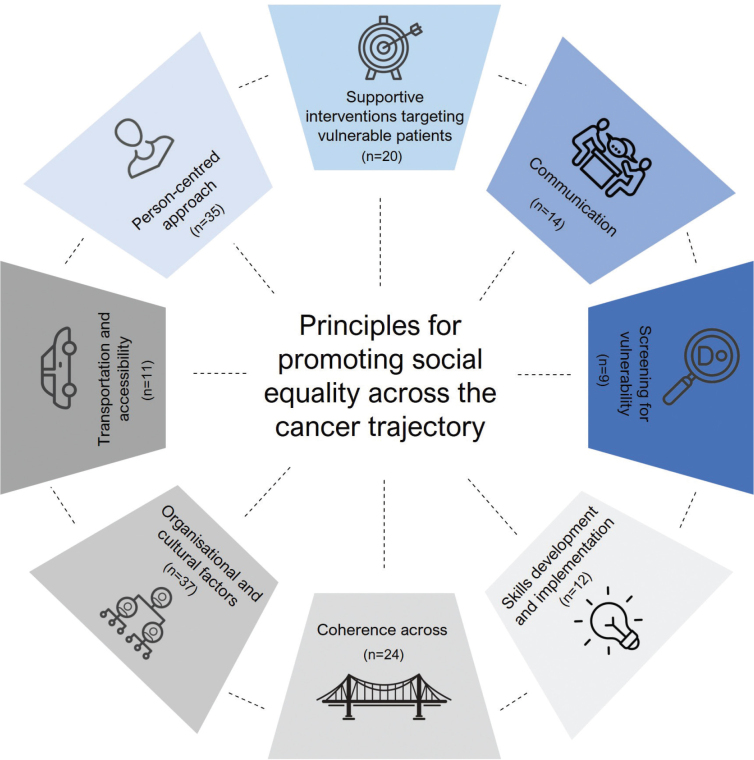
The eight guiding principles for promoting social equality across the cancer trajectory. Principles on patient-provider level are colored blue and those on organisational and political level are colored grey. n represents the total number of ideas in the principle.

The calculation of the median importance ratings for the final clusters showed that all clusters were rated equally, each receiving a median score of 3. (See Supplementary file 1 for details on each cluster, including the median importance rating for each idea as well as for the clusters overall.) The final eight clusters, shown in Figure 2, reflect the principles for promoting social equity in the cancer trajectory and are grouped into two levels: (1) Patient–provider and (2) organizational–policy.

The first cluster, *Person-centred approach*, advocates tailoring health care to vulnerable patients’ bio-psycho-social profiles, including life context, needs assessment, and early identification of resources. Discussions highlighted positive experiences with Smart Phrases in electronic medical records and stressed that person-centred care requires time and stratified approaches.

*Building a close relationship is absolutely central to the patient-centred approach. You need to invest a lot of time and resources into building that relationship because, without it, you can’t understand where the patient is and what their need is.*

The second cluster, *Supportive interventions targeting vulnerable patients*, focuses on strategies to assist vulnerable patients across their cancer journey. Suggestions included assigning navigators or coordinators to support care continuity, arranging transport, managing appointments, ensuring access to rehabilitation, and facilitating communication across sectors. A researcher further elaborated on added benefits of a dedicated nurse for vulnerable patients.

*The fact that the patient has a dedicated nurse navigator who knows the patient very well is one of the positive experiences we’ve had from the NAVIGATE project. This allows for a quick response to physical and psychological symptoms because the nurse is familiar with the patient.*

Peer support programs, where patients are matched with peers from a non-governmental organization were also suggested as a potential intervention to support vulnerable patients. However, discussions stressed that such interventions must be offered systematically to all vulnerable patients.

*Yes, it works if you do it systematically; otherwise, it becomes unequal. Then it’s those who shout the loudest who get it.*

The third cluster, *Communication*, highlights the fact that engaging with vulnerable patients requires time, trust, and specialized communication skills. Both ideas and discussions emphasized the importance of building a close, trusting relationship and providing healthcare professionals with training on how to communicate effectively with this group.

*Meeting a vulnerable patient requires training in communication, but with this patient group, it also requires time, time to build a relationship in order to achieve effective communication.*

Communication mode should be flexible offering in-person, phone, or online options based on patient preferences. Information must be clear, jargon-free, and supported by visuals or videos to aid understanding. Discussions also stressed that directing patients to websites or apps may be ineffective for socially vulnerable individuals.

*You need to be careful when saying, ‘You can read about it on this website or download this app’. This approach doesn’t work for socially vulnerable individuals because they often aren’t able to follow through.*

Lastly, the ideas pointed out that professionals should consider differences in digital and health literacy, tailoring communication accordingly.

The fourth cluster, *Screening for vulnerability*, recommends systematic vulnerability screening during diagnosis and treatment to tailor care pathways. As one participant noted.

*If we can clarify who they are, we can better focus on it. Some actions might be done intuitively, while others may require specific guidelines. But simply raising awareness is already a step in the right direction.*

Consistency was strongly emphasized: all patients should be screened systematically to avoid reinforcing inequalities. A participant warned.

### What’s important is that it’s systematic; otherwise, it could potentially lead to increased inequality

The NAVIGATE project within COMPAS showed how screening can prevent patients from slipping through healthcare gaps. Discussions favoured patient-reported outcomes over broad categories like income or education, which may not reflect individual needs. For instance, someone with low education might still be health-literate. PRO-based assessments targeting barriers such as access or comprehension were preferred, with consensus on the need for validated screening tools.

The fifth cluster, *Skills Development and Implementation,* the importance of training healthcare professionals to engage effectively with socially vulnerable patients and reduce stigma. Participants also strongly advocated for hiring more nurses specifically trained to address social needs. Additionally, several noted that many current interventions fail to reach the most vulnerable, highlighting the need for implementation research on how to adapt successful interventions for this group.

*In several of the intervention studies conducted, we simply can’t reach the socially vulnerable. So, when we now find that the intervention itself works, we need to look at how we can also extend its reach to the vulnerable. We obviously need to be better at considering this issue right from the start of the research process.*

The sixth cluster, *Coherence across*, calls for better cross-sectoral coordination, particularly between hospitals and primary care. This includes optimizing cross-sectoral workflows, clarifying responsibilities, ensuring access to medical journals across sectors, and increasing knowledge and awareness among hospital staff regarding interventions and possibilities in the primary healthcare sector. Specifically, the gaps in referrals from hospitals to primary care due to limited awareness or trust in community services were mentioned.

*Hospital departments are often inadequate at referring patients to the existing primary care services, because they are not fully aware of the services and capabilities of primary care providers and thus lack trust in them.*

The seventh cluster, *Organisational and cultural factors*, highlights the need for structural changes to accommodate the needs of vulnerable patients. These include allocating sufficient time and resources, extended consultations, and stratified follow-up. One participant noted:

*The expensive part of cancer treatment is the treatment itself, so if you’re not willing to pay for additional consultations for vulnerable patients, is it even worth starting such an expensive treatment? If you begin treatment, you need to make it possible for them to complete it, which requires extra support for the vulnerable group.*

Another person added:

*You need to spend time identifying the barriers that prevent them from not completing the treatment.*

The findings also pointed to the need for a cultural paradigm shift, including a stronger focus on quality of life and early preventive efforts, rather than solely on survival metrics.

The eighth cluster, *Transportation and Accessibility*, highlights the crucial role of transport in accessing cancer care. Participants emphasised that the ability to attend appointments often hinges on whether transportation is affordable and accessible. While financial support for transport expenses was widely recommended, discussions revealed that this alone may be insufficient for socially vulnerable individuals.

*In several of the intervention studies we find that we cannot reach the most socially vulnerable, even when transportation costs are reimbursed. We can’t buy them a car, and many lack a support network to help them get to appointments.*

As a result, more flexible models such as home-based care were proposed. However, opinions on this solution varied. Some viewed it as a way to improve access, while others cautioned that it could inadvertently place additional responsibility on vulnerable patients.

*From our studies, I’ve seen that home treatment can make patients even more vulnerable when they are left to manage treatment alone at home – especially the most vulnerable.*

This underscores the need for tailored transport and care delivery solutions that consider both structural and individual-level barriers.

## Discussion

The novelty of this study lies in its synthesis of cross-sectoral, practice-based knowledge from COMPAS, principles spanning the cancer continuum and addressing both individual and systemic factors. Unlike prior work focused on specific phases or settings, this integrated framework is grounded in empirical evidence and expertise, generated through GCM. The principles emphasize addressing inequality at multiple levels, from patient, provider interactions to political structures, aligning with recommendations for reducing disparities in cancer and other chronic conditions [26, 27]. Their interrelated nature underscores the complexity of the challenge, requiring multilevel, coordinated strategies involving diverse stakeholders [26, 28].

Findings, in line with existing literature, highlight the dependency of person-centred care on healthcare professionals’ interpersonal skills [29]. This is particularly critical for patients with low SEP, who are less likely to voice concerns or participate actively [30, 31]. Hence, providers must adopt proactive approaches to integrate patient preferences into decision-making.

Effective communication is central to person-centred care and plays a crucial role in shaping both patient experiences and health outcomes [29]. Participants stressed the need for clear, jargon-free information supported by visuals to reach all literacy levels. Importantly, this is not unique to cancer care. A Danish population-based survey of 29,000 patients across various conditions showed that lower educational groups struggle most with health information, though cancer patients generally display higher health literacy than those with other chronic conditions [32].

Another guiding principle was proving supportive interventions specifically targeted at vulnerable patients, such as patient navigation. Within the COMPAS, the NAVIGATE project assigns nurse navigators to vulnerable lung cancer patients, providing up to 12 months of support [33]. Also, evidence outside COMPAS supports the effectiveness of such targeted support. Similar interventions have improved initiation and adherence to therapies in other cancer types, such as breast cancer [34–37]. In addition, combining nurse navigation with a person-centred approach appears especially beneficial [29].

Our study highlights the importance of implementing a systematic approach to vulnerability screening early in the cancer trajectory to ensure provision of tailored, specialized care to those who need it most. Current tools, such as the Geriatric 8 and Clinical Frailty Scale [38, 39], have effectively primarily assessed physical frailty in oncology settings, but broader measures are needed to better assess vulnerability across various domains [40]. Efforts within COMPAS to develop a register-based Social Vulnerability Index (rSVI) and patient-reported screening tools are promising, though further validation is required [33, 41].

The study findings stress that training healthcare professionals to recognize and respond to social inequality is vital; however, this is underexplored. A systematic review of 31 interventions to improve cancer care for socially disadvantaged groups found that only one included provider education [34]. Future research should focus on integrating social equity content into healthcare education using divers teaching strategies, such as curricular initiatives, learning strategies, university programs, and civil society initiatives programs [27, 42].

Improving accessibility to healthcare emerged as another principle in this study. This finding aligns with existing research showing that geographical access remains a barrier, particularly for rural patients [43, 44]. Despite evidence of its impact, few interventions explicitly address this issue [34]. Our study may inform how to address accessibility barriers among geographically and socially disadvantaged populations. Interestingly, digital health (eHealth) was not a prominent theme, perhaps reflecting concerns that such technologies may inadvertently widen inequalities if vulnerable populations lack digital access or skills [45, 46].

These principles are relevant for multiple stakeholders. For patients, they offer more equitable, person-centred care. For professionals, they underscore the need for inclusive communication and tailored interventions. Societally, they provide a framework for reducing barriers and informing equity-focused policy. Finally, they lay a foundation for research on implementing and evaluating equity-oriented cancer care.

### Strengths and limitations

This study synthesizes findings from several projects and programmes, providing a broader understanding compared to individual studies alone. By integrating diverse methodologies, professional backgrounds, populations, and contexts, the study enhances validity. The inclusion of qualitative data from idea generation and cluster validation further enriched insights into the rationale behind proposed ideas.

However, the study is limited by its primary reliance on Danish research, which may restrict international transferability. The initial COMPAS research also lacked an explicit focus on ethnicity and migrant health, both central to understanding health disparities [47]. Ongoing COMPAS studies now address inequalities in cancer outcomes and experiences of care among patients born outside Denmark. Finally, the validation meeting within the GCM approach led to relocating over half of the ideas, an unusual outcome that questions the validity of the original sorting. This likely reflects the wide diversity in participants’ interpretations and underscores the complexity of collective meaning-making.

## Conclusions

This study finds that promoting social equity in cancer trajectories requires actions at the patient–provider, organisational, and policy levels. Key principles include person-centred care, targeted support for vulnerable patients, improved communication, and systematic screening for vulnerability. Additionally, promoting equity involves investing in skills development and implementation studies, fostering cross-sector coherence, cultivating inclusive organizational cultures, and enhancing the accessibility of healthcare services.

## Supplementary Material



## Data Availability

Data can be shared upon request to the corresponding author.
