# Supplementary material for: Principles to promote social equality across the cancer trajectory: A group concept mapping study
Source: Acta Oncol. 2025 Nov 19;64:44738. doi: 10.2340/1651-226X.2025.44738 (PMC12645219; doi:10.2340/1651-226X.2025.44738)
Supplement: Supplementary file 1 [file AO-64-44738-s1.pdf]

Supplementary material has been published as submitted. It has not been copyedited, or typeset by Acta Oncologica

Supplementary file 1 – Clusters and ideas

1 Person-centred approach (n=35): Median 3 (3-4)

Idea number	Ideas	Median of ideas
2	Stratified care	3
5	Involvement of relatives	3
28	Do not make multiple demands on patients to change their lifestyle	3
36	Patient-centred approach in all treatment and care	4
29	Involve network	3
41	Training can/should be re-thought and conducted on the patient's terms	3
42	Understanding that the patient may not want the medically most optimal treatment	3
43	Ensure follow-up on identified needs	4
45	Be aware that even the least resourceful patients actually have resources	3
47	Expand personalised medicine to personalised rehabilitation	3
51	Be aware that patients and any accompanying relatives do not agree	3
52	Handling that patients and any relatives do not agree	3
65	Patients should not be treated the same	3
67	High degree of flexibility in intervention components when possible	3
69	That the healthcare professional understands that the cancer disease may not be the 1st priority/biggest problem for the patient	3
72	That the healthcare professional cares about/asks about the patient's overall health	4
73	That the healthcare professional cares about/asks about the patient's social situation	3
74	Nurses need to be open to not just focusing on managing chemotherapy	3
75	Nurses must be open to focusing on all aspects of the patient's life	3
84	Accommodate that there are other issues than cancer at stake	3
88	Involve relatives	3
92	Respond to needs in a timely manner	3
94	Differentiated treatment	4
103	Focus on the patient as a whole person	4
106	Offer interventions aimed at lifestyle risk behaviours (KRAM – Diet, Smoking, Alcohol, Exercise)	3
114	Flexibility and adaptation of treatment	3

119	By adapting treatment to the patient’s physical, psychological, social needs	3
124	Mapping patient profile from bio-psycho-social factors	3
125	Ensure identification of needs during and at the end of treatment	3
127	Assess the patient’s needs	4
132	Involve the patient’s resources in the planning of treatment	3
133	Attention to patients’ social issues when we meet them at the hospital	3
135	An adequate conversation about the patient’s wishes and needs	3
139	Early detection of patient resources in the cancer treatment	3
152	Respect for patient choices and opt-outs	4

2 Communication (n=14): Median 3 (3-4)

6	Information material should be easy to understand without too many specialised terms	3
7	Information material should contain short sentences	3
8	Information material should contain pictures and illustrations	3
13	Flexibility in relation to the patient’s preferred mode of communication (in person, online, telephone)	3
16	Understanding of different IT competences/e-health competencies	3
30	Develop easily understandable information, e.g. videos	3
31	Motivational interview	3
35	Patient-centred approach in all communication	3
56	Keep the ‘quiet’ patients in mind	3
63	More interventions in the short term (e.g. short phone conversations with nurses) can be worthwhile in the long run	3
66	Dare to have the difficult conversations	3
91	Ask the patient: What is most important to you?	4
111	Nurse hotline so patients can get answers to questions that arise along the way	3
113	It is necessary for staff to build a close and safe relationship with the patient	3

3 Supportive services targeted at the vulnerable (n=20): Median 3 (3-4)

18	Buddy schemes for vulnerable patients	3
21	Patient-peers	3
64	Actively assist the patient in their contact with the health/rehabilitation services available in each municipality	3
81	Focus on improving health behaviours among socially disadvantaged patients	3
87	Helping disadvantaged/vulnerable patients remember healthcare appointments	3
89	Provide support to socially vulnerable patients	3

93	As few healthcare professionals in each course of treatment as possible	3
95	Give more support to those with the greatest need	3
105	Focus on accessibility of healthcare for vulnerable patients	3
121	Dedicated nurse who follows the patient throughout the course of treatment	3
128	If the patient is accompanied either by relatives or by a professional assistant (e.g. nurse)	3
136	Optimise the support in the form of a navigator and coordinator acting as a conversation partner	4
137	Make patients aware of the possibility of the service from Social Sundhed (Social Health) if support and understanding is needed	3
138	Optimise the course of treatment with a contact person if necessary, so that the patient is not lost during the transitions between sectors	3
143	Focus on health literacy among the socially disadvantaged	3
147	Develop forms of user involvement specifically suited to patients/people in vulnerable positions	3
153	Optimise the support in the form of a navigator and coordinator who can participate in conversations	3
159	Optimise the support in the form of a navigator and coordinator who can help with transportation for various examinations, treatments, etc.	3
160	Optimise the support in the form of a navigator and coordinator who can support the patient throughout the course of treatment	3
162	Optimise the support in the form of a navigator and coordinator that can act as a link between sectors	3

4 Vulnerability screening (n=9): Median 3 (3)

49	Vulnerability screening should be based on patient-reported questions	3
50	Vulnerability screening should not be based only on the doctor’s assessment	3
55	Systematic screening for symptoms during treatment to avoid toxicity and discontinuation of treatment	3
71	Develop screening tools (validated) to identify vulnerable patients/citizens	3
80	Focus on screening tools to identify particularly vulnerable people at the start of the course of treatment	3
82	Screen for vulnerability at diagnosis and during treatment	3
117	Screening the patient for lifestyle risk behaviours (KRAM – Diet, Smoking, Alcohol, Exercise)	3
118	Screening of vulnerable patients to optimise the course of treatment	3
126	Screening for vulnerability at the time of diagnosis	3

5 Skills development and implementation (n=12): Median 3 (2-4)

12	Increased focus on implementation studies	2
17	Training of health professionals in social inequality/social vulnerability	3
39	Include education on social inequality in health in doctors’, nurses’ etc. education programmes	3

44	More feasibility studies before it is attempted to implement an intervention in practice	2
46	Teach staff about the consequences of social inequality for their patients	3
68	Teach staff to involve patients with fewer resources when there is a need for joint decision-making	3
123	When we have effective interventions from research that improve cancer treatment, it is important to ensure their successful implementation	3
129	Training staff in communication	3
140	Ensure that healthcare professionals have the skills to deal with patients with fewer resources	4
154	Ensure that healthcare professionals have the skills to assess which initiatives the individual may benefit from	4
157	Learn from those who are hard to reach/who don't make use of the offers of the healthcare system	3
161	Increase knowledge and make it clearer that everyone gets to know citizens early in the process, so that early interventions can be carried out	3

6 Cross-sector coherence (n=24): Median 3 (3-4)

9	Shared patient records across sectors	3
10	Better cross-sectoral knowledge sharing	3
11	A more coherent patient course	4
14	Same IT systems/journal access across regions	3
23	Work with cross-sectoral workflows so that responsibility is clear and the baton is passed on unambiguously	4
24	Work to ensure that the baton is passed on unambiguously	3
25	Strengthen the patient's experience of coherence in the course of treatment	3
26	Continuity in relation to which professionals are involved in the patient's course of treatment	3
32	Ensure better co-ordination across healthcare system and municipality	3
33	Ensure better collaboration across healthcare system and municipality	3
37	Follow-up from the place of treatment	3
53	Optimise cross-sectoral co-operation	4
57	Building bridges between sectors	3
61	Use the primary sector's resources	3
85	Co-operation with social nurses	3
99	Trust in primary sector partner	3
107	Consistency in interdisciplinary communication	3
110	Summary for GP in case of worsening of condition	3
112	Involve experts from other specialties in MDT to manage comorbidity as best as possible	3
122	Ensuring intersectoral communication about the patient	3
141	Interdisciplinary approaches at multiple levels of the healthcare system	3

148	Focus on organisational frameworks and transitions	3
150	Modify incentive structures so that they promote co-operation between general practice and the secondary sector, for the benefit of the patient	3
158	Increase knowledge and awareness of efforts across sectors	3

7 Organisational and cultural factors (n=37): Median 3 (2-4)

1	Set up a dedicated team of doctors and nurses specially trained in vulnerable patients	3
3	Stratified follow-up	3
4	Spend more resources on vulnerable patients	3
15	Every time a new practice is introduced in healthcare, the risk of it increasing or compensating for inequality should be assessed – just as all political bills must include a CO_2 report	2
19	Employ more doctors and nurses	3
20	More time for conversation/relationship formation/individual needs	3
22	Increase social worker resources	3
27	Greater inclusivity in healthcare with regard to socially marginalised people	4
34	Devote extra time to socially disadvantaged patients	4
54	Employing more social nurses	3
60	Think about prevention already during childhood	3
62	Prioritise assessment of needs from department management	3
70	Ensure that healthcare professionals have time for the most vulnerable patients	3
76	Interventions are offered with great flexibility with regard to time	3
78	Find out if someone is to receive more – who can then get less in a responsible manner	3
79	First of all, make structural changes that counteract social inequality	3
86	More focus on the importance of patient-responsible doctor and nurse	3
96	Scheduling extra time for vulnerable and disadvantaged patients	4
97	Organisational changes to ensure that vulnerable patients are allocated sufficient time	4
98	Cultural changes to ensure that vulnerable patients are allocated time	3
100	Plenty of time for conversation	4
101	Plenty of time for follow-up	3
108	Reduce stigma	3
109	Reduce discrimination	3
116	Allow extra time for the patient who has difficulty keeping up	3
120	Change the paradigm from not only focusing on survival to also focusing on quality of life	3
130	Targeted screening campaigns for socially vulnerable groups who do not participate as much in cancer treatment	3

131	More uniform cancer rehabilitation services (especially physical training) in all municipalities	3
134	More time for the socially disadvantaged people	3
142	Apply a syndemic theory perspective and look at the multifactorial drivers that perpetuate social inequality	2.5
144	Organisational development that learns from patients/people in vulnerable positions	3
145	Organisational development that learns from previous experiences with assessment of needs	3
146	Common professional and financial framework for regions, general practices and municipalities	3
149	Focus on practices that create inequality in clinical everyday life	4
151	Provide the option to have an extended consultation with their own doctor for patients with cancer who are socially disadvantaged, have complex problems and/or have multiple diseases	3
155	Interdisciplinary interventions throughout the life course	3
156	Focus on cultural health capital in municipalities	3

8 – Transportation and accessibility (n=11): Median 3 (3)

38	Ensure good and stable transportation of patients to and from treatment	3
40	Training programmes need to be re-thought and can take place at home	3
48	More treatment in own homes for socially vulnerable patients	3
58	Transportation assistance for vulnerable/disadvantaged patients	3
59	Subsidies for transportation costs for vulnerable/disadvantaged patients	3
77	Interventions are offered with great flexibility with regard to location	3
83	Offer treatment at home instead of transporting the patient to the hospital	3
90	Secure means for patients to be transported to places of treatment	3
102	Move the offer of treatment closer to the patient if possible	3
104	Focus on transportation for healthcare services for vulnerable patients	3
115	Subsidies for transportation to physiotherapy (currently not covered by FlexTrafik)	3
